# Genetic correlation of the plasma lipidome with type 2 diabetes, prediabetes and insulin resistance in Mexican American families

**DOI:** 10.1186/s12863-017-0515-5

**Published:** 2017-05-19

**Authors:** Hemant Kulkarni, Manju Mamtani, Gerard Wong, Jacquelyn M. Weir, Christopher K. Barlow, Thomas D. Dyer, Laura Almasy, Michael C. Mahaney, Anthony G. Comuzzie, Ravindranath Duggirala, Peter J. Meikle, John Blangero, Joanne E. Curran

**Affiliations:** 1 South Texas Diabetes and Obesity Institute, University of Texas Rio Grande Valley School of Medicine, Brownsville, TX 78520 USA; 2 Baker IDI Heart and Diabetes Institute, Melbourne, VIC Australia; 3 Department of Genetics, Texas Biomedical Research Institute, San Antonio, TX USA

**Keywords:** Type 2 diabetes, Plasma lipidome, Genetic correlation, Bivariate trait analyses, Family studies

## Abstract

**Background:**

Differential plasma concentrations of circulating lipid species are associated with pathogenesis of type 2 diabetes (T2D). Whether the wide inter-individual variability in the plasma lipidome contributes to the genetic basis of T2D is unknown. Here, we investigated the potential overlap in the genetic basis of the plasma lipidome and T2D-related traits.

**Results:**

We used plasma lipidomic data (1202 pedigreed individuals, 319 lipid species representing 23 lipid classes) from San Antonio Family Heart Study in Mexican Americans. Bivariate trait analyses were used to estimate the genetic and environmental correlation of all lipid species with three T2D-related traits: risk of T2D, presence of prediabetes and homeostatic model of assessment – insulin resistance. We found that 44 lipid species were significantly genetically correlated with one or more of the three T2D-related traits. Majority of these lipid species belonged to the diacylglycerol (DAG, 17 species) and triacylglycerol (TAG, 17 species) classes. Six lipid species (all belonging to the triacylglycerol class and containing palmitate at the first position) were significantly genetically correlated with all the T2D-related traits.

**Conclusions:**

Our results imply that: a) not all plasma lipid species are genetically informative for T2D pathogenesis; b) the DAG and TAG lipid classes partially share genetic basis of T2D; and c) 1-palmitate containing TAGs may provide additional insights into the genetic basis of T2D.

**Electronic supplementary material:**

The online version of this article (doi:10.1186/s12863-017-0515-5) contains supplementary material, which is available to authorized users.

## Background

There is a strong genetic basis to the risk of developing type 2 diabetes (T2D) with heritability estimates approximating 60% [1, 2]. In the continued quest for early identification of T2D, it is important to find biomarkers that, at least partly, explain the genetic basis of T2D pathogenesis. Concentrations of specific plasma lipid species are considered to be potential biomarkers of type 2 diabetes (T2D) because subtle changes in the plasma lipidome are associated with the risk of diabetes [3, 4], prediabetes [4] and insulin resistance [5, 6]. Further, the overall heritability of the plasma lipidome is 37% with many lipid species individually showing significant heritability [3]. Together, these observations raise an interesting possibility of an overlap (i.e. pleiotropy) in the genetic basis of the plasma lipidome and T2D related traits. Evidence of such shared genetic bases can lend more credence to the candidature of plasma lipidome as a biomarker of T2D. Direct evidence from human studies to support or refute this conjecture is currently not available. Here, we report our findings from the large study of extended Mexican American families – the San Antonio Family Heart Study (SAFHS) – to investigate the shared genetic basis of plasma lipid species concentrations and three phenotypic traits related to T2D in the high risk population of Mexican Americans.

## Methods

### Study participants

Data for this study came from the 1202 SAFHS individuals (representing 42 families) on whom plasma lipidomic data as well as other phenotypic data was available. Details of the study participants have been described elsewhere [7]. We used the phenotypic data collected at baseline as well as during an average of 9250.64 person-years of follow-up through a maximum of 4 clinic visits (1 baseline visit and 3 additional follow-up visits) spaced approximately 5 years apart. Informed consent was obtained from all participants before collection of samples. The Institutional Review Board of the University of Texas Health Science Center at San Antonio approved the study. The biological relationships observed in the study sample are shown in (Additional file 2: Table S1).

### T2D-related traits

We studied three traits related to T2D: risk of T2D, presence of prediabetes and homeostatic model of assessment – insulin resistance (HOMA-IR). Risk of T2D was used as a discrete trait and was coded as 0 if an individual did not have T2D at baseline and remained T2D-free throughout follow-up. If the individual either had T2D at baseline or was detected as a new case of T2D during follow-up the individual was considered to have T2D and was coded as 1. Presence of T2D was diagnosed [8] as presence of at least one of the following: i) self-reported T2D; ii) fasting blood glucose ≥126 mg/dl and/or 2-h post glucose load blood glucose ≥200 mg/dl or; iii) history of anti-diabetic medication use. The other two traits (presence of prediabetes and HOMA-IR) were studied in only those individuals free of T2D at baseline. Prediabetes was defined as presence of impaired fasting glucose (IFG) or impaired glucose tolerance (IGT) or both. IFG was defined as fasting blood glucose levels of 100–125 mg/dl while IGT was defined as blood glucose levels of 140–199 mg/dl after 2-h of 75 g oral glucose load. Finally, insulin resistance was quantified using HOMA-IR which was estimated as follows – fasting blood glucose (mmol/L) x fasting plasma insulin (μU/ml)/22.5 [9].

### Phenotypic assessments

We assayed 319 lipid species representing 23 lipid classes. This assessment was only done at the baseline visit. Concentration of the lipid species in the plasma was measured using a combination of high performance liquid chromatography and mass spectroscopy as described elsewhere [4, 10]. Briefly, 10 μL aliquots of plasma were combined with 200 μL CHCl_3_/MeOH (2:1) and 15 μL of internal standard mix. Then samples were mixed, sonicated and allowed to stand (20 min) at room temperature. Mass spectrometric analysis was performed using on extracted lipid injections. Identification and quantitation of lipid species was performed by liquid chromatography electrospray ionisation-tandem mass spectrometry using an Applied Biosystems 4000 QTRAP. Liquid chromatography was performed on a Zorbax C18, 1.8 μm, 50 × 2.1 mm column at 300 μL/min. Quantification of individual lipid species was then performed using scheduled multiple-reaction monitoring (MRM) in positive ion mode [11, 12]. Lipid concentrations were calculated by relating the peak area of each species to the peak area of the corresponding internal standard. Cholesterol ester species were corrected for response factors determined for each species. Lipidomic profiling studies were conducted in the Metabolomics Laboratory, Baker IDI Heart and Diabetes Institute. Full details of the methods used to assess other clinical phenotypic traits like waist circumference, blood pressure and biochemical phenotypes have been described previously [13].

### Statistical analyses

Since the focus of this investigation was pleiotropy between T2D-related traits and the plasma lipidome, we used bivariate trait analyses within the variance components framework [14]. Variance components methods hypothesize that the total phenotypic variance can be partitioned into genetic and residual environmental variance for a given trait. When two traits are being simultaneously considered (bivariate analyses), the phenotypic covariance between the two traits is considered to be a function of the variance of each trait, the heritability of each trait and the genetic and environmental correlation between the traits. Specifically, the phenotypic correlation between the two traits ρ_P_ is defined as follows:

$$ {\rho}_P\left( i, j\right)={\rho}_G\left( i, j\right)\sqrt{h_i^2+{h}_j^2}+{\rho}_E\left( i, j\right)\sqrt{\left(1-{h}_i^2\right)+\left(1-{h}_j^2\right)} $$

In this equation, i and j are indexes for the two traits being considered, h^2^ is the heritability ρ_G_ is the genetic correlation coefficient, and ρ_E_ is the environmental correlation coefficient. Maximum likelihood methods are then used to arrive at the estimate of ρ_G_ and ρ_E_. Statistical significance for a null hypothesis that ρ_G_ = 0 was tested by constraining ρ_G_ to 0 and using a chi-square test based on the difference in the log-likelihood of the unconstrained and constrained model.

To account for multiple comparisons, we used the Benjamini-Hochberg method of controlling for false discovery rate [15]. To investigate whether significant proportion of lipid species within a class were genetically correlated with a given trait, we used the Fisher’s test *p* value and converted it into an enrichment score (ES) using a negative logarithmic transformation. We used the well-established SOLAR software [16] to conduct the bivariate analyses and estimate the genetic correlation coefficient between each T2D-related trait and each lipid species. We also tested the hypothesis that the circulating levels of lipid species were influenced by use of lipid-lowering medications. For this we used censored normal regression [17] where each lipid species concentration was used as the dependent variable, a two-level indicator variable was used as the predictor variable and the standard errors were robustly estimated by using the family identifier as a clustering variable. The censored normal regression analyses were conducted using Stata 14.0 software (Stata Corp, College Station, TX). Statistical significance for all the analyses was tested at a global type I error rate of 0.05.

## Results

### Study participants

This study included a total of 1202 individuals of Mexican American descent representing 42 extended families. The mean age of the participants was 39.36 years (standard deviation (16.87) and 724 (60.23%) were females. At the time of enrolment, 1.84 and 9.62% individuals were receiving lipid-lowering and anti-hypertensive agents, respectively. Results of the censored normal regression analyses (see Additional file 3: Table S2) showed that the influence of lipid-lowering medications on individual lipid species was statistically non-significant. At baseline 176 (14.64%) individuals had T2D while another 124 (10.32%) developed T2D during follow-up. Together, 300 (24.96%) individuals had T2D either at baseline or during follow-up. Individuals who were free of T2D at baseline (*n* = 1026) were used for data on prediabetes (as discrete trait) and HOMA-IR (as continuous trait). All the T2D-related traits were significantly and substantially heritable (risk of T2D: h^2^r = 0.6444, *p* = 5.68 × 10^−13^; presence of prediabetes: h^2^r = 0.4321, *p* = 0.0001; and HOMA_IR: h^2^r = 0.4155, *p* = 1.90 × 10^−17^).

### Genetic correlation between T2D-related traits and lipid species

**Fig. 1:**
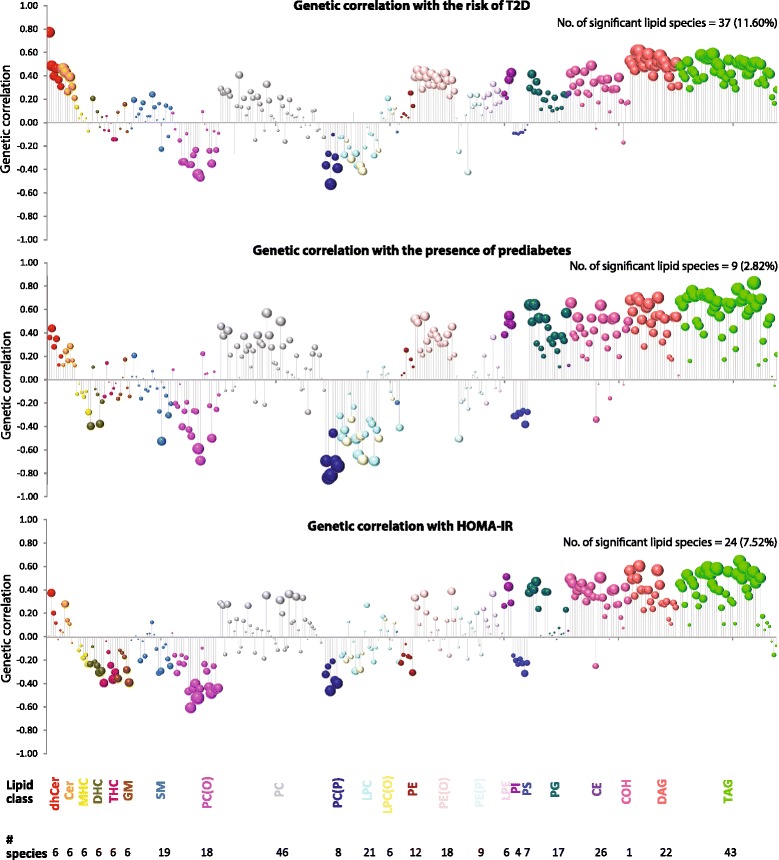
Genetic correlation of each lipid species with T2D-related traits: risk of T2D, presence of prediabetes and homeostatic model of assessment – insulin resistance (HOMA-IR). Risk of T2D and presence of prediabetes were modeled as discrete tests using the liability threshold approach while HOMA-IR was used as a continuous, inverse-normalized trait. Presence of prediabetes and HOMA-IR analyses were restricted to individuals who did not have T2D at baseline (*n* = 1026). Plots show bubble charts with the lipid species on the abscissa and the estimated genetic correlation coefficient (ρ_G_) on the ordinate. The size of the bubble is proportional to –log_10_p, where P is the statistical significance to test the null hypothesis that ρ_G_ = 0. The number of lipid species that were significantly genetically correlated (after controlling for false discovery rate) are shown at the upper-right corner of each plot. The bubbles are color coded to indicate lipid classes shown at the bottom of the Figure. The lipid classes studied were: dihydroceramide (dhCer), ceramide (Cer), monohexosylceramide (MHC), dihexosylceramide (DHC), trihexosylceramide (THC), GM3 ganglioside (GM), sphingomyelin (SM), phosphatidylcholine (PC), alkylphosphatidylcholine (PC(O)), alkenylphosphatidylcholine (PC(P)), lysophosphatidylcholine (LPC), lysoalkylphosphatidylcholine (LPC(O)), phosphatidylethanolamine (PE), alkylphosphatidylethanolamine (PE(O)), alkenylphosphatidylethanolamine (PE(P)), lysophosphatidylethanolamine (LPE), phosphatidylinositol (PI), phosphatidylserine (PS), phosphatidylglycerol (PG), cholesteryl ester (CE), cholesterol (COH), diacylglycerol (DAG) and triacylglycerol (TAG)

**Fig. 2:**
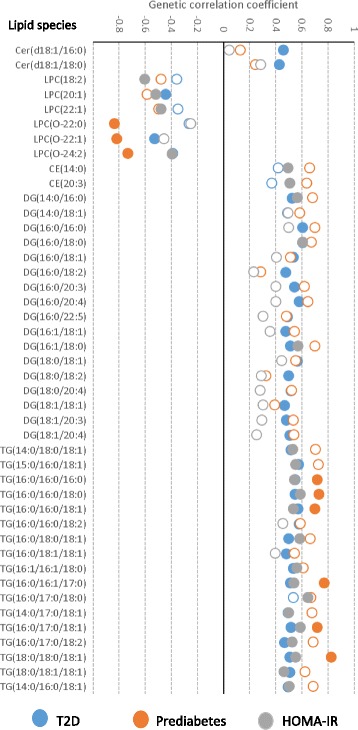
Comparison of the significantly associated lipid species with differing T2D-related traits. The plot shows estimated genetic correlation coefficients for each the 44 lipid species that were associated with at least one T2D-related trait. Filled circles indicate statistically significant associations (after accounting for multiple comparisons) while hollow circles indicate non-significant associations. The markers are color-coded for each trait as indicated in the key

We first conducted the bivariate trait analyses for all combinations of T2D-related traits and lipid species. The results of these analyses are summarized in Fig. 1 and detailed in (Additional file 4: Table S3). We observed that after correction for multiple comparisons 37 (11.58%), nine (2.82%) and 24 (7.52%) lipid species remained significantly genetically correlated with the risk of T2D, presence of prediabetes and HOMA-IR, respectively. Overall, there were 44 (13.79%) lipid species (shown in Fig. 2) that were significantly correlated with at least one of the three T2D-related traits. A comparison of the trait-specific associations indicated that i) most of the genetic correlations with T2D were in 0.4–0.6 range; ii) stronger (albeit less frequent) genetic correlations were found with prediabetes and iii) similar patterns of genetic correlations were observed for T2D and HOMA-IR even though the analyses for HOMA-IR were restricted to individuals who did not have T2D at baseline. Of the 44 lipid species, the following six lipid species were significantly genetically correlated with all three T2D-related traits: TG(16:0/16:0/16:0), TG(16:0/16:0/18:0), TG(16:0/16:0/18:1), TG(16:0/16:1/17:0), TG(16:0/17:0/18:1) and TG(16:0/18:0/18:1) indicating a preponderance of 1-palmitate in these TAG species.

### Comparison of genetic and environmental correlations

Interestingly, significant shared environmental influences were observed for 114 (35.7%), 59 (18.5%) and 172 (53.9%) lipid species with T2D, prediabetes and HOMA-IR, respectively (Additional file 5: Table S4). However, the strength of environmental correlation was generally lesser than that for corresponding genetic correlation. As shown in Table 1, the dihydroceramide/ceramide species showed the strongest environmental correlations while the LPC and some PC species showed a strong negative environmental correlation. In all there were 48 lipid species that were environmentally correlated significantly with all three traits.

**Table 1:** Environmental correlation coefficients of lipid species for indicated T2D-related traits^*^

	Risk of T2D	Prediabetes	HOMA-IR
Highest ρ_E_	Cer(d18:0/24:1), 0.3177, *p* = 2.7 × 10^−8^	PS(40:5), 0.2917, *p* = 1.0000	Cer(d18:0/22:0), 0.4234, *p* = 2.3 × 10^−24^
Lowest ρ_E_	LPC(26:0), −0.3588 *p* = 0.0001	PC(39:6), −0.2076, *p* = 0.1501	PC(P-34:1), −0.2972, *p* = 7.8 × 10^−17^
N (significant)	114	59	172

^*^cells for the first two rows contain name of the lipid species, the observed ρ_E_ and its significance value

For the lipid species that showed significant genetic correlation with at least one T2D-related trait (*n* = 44), we found that the genetic correlation was generally stronger than the environmental correlation (see Additional file 1: Figure S1). Specifically, only 1 (Cer(d18:1/16:0)) and 4 (Cer(d18:1/16:0), DG(16:0/18:2), DG(18:0/18:2) and DG(18:1/20:4)) lipid species showed a larger environmental correlation as compared to genetic correlation when investigated in the context of prediabetes and HOMA-IR, respectively – all the remaining combinations of the 44 lipid species and 3 T2D-related traits showed a larger genetic than environmental correlation.

### Genetic correlation at the level of lipid classes

**Fig. 3:**
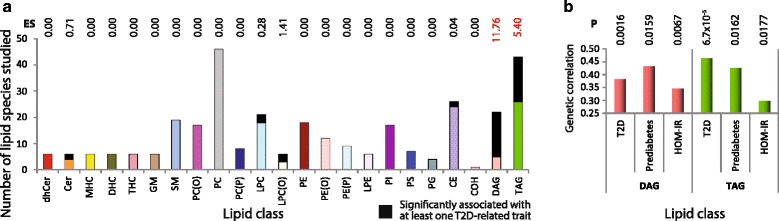
Genetic correlation of lipid classes with traits related to type 2 diabetes (T2D). **a** Bar chart showing the number of species studied within each lipid class and the number of statistically significant (after correction for false discovery rate) lipid species within each class. The statistically significant species are shown as *black* component within each color coded lipid class. Numbers above each bar indicate the enrichment score (ES) for the given lipid class. ES more than 2.66 showed statistically significant enrichment (shown using *red colored numbers*) in the genetically correlated species for a given lipid class. **b** Genetic correlation of the DAG and TAG lipid classes with T2D-related traits. Details of the T2D-related traits are as described in legends for Fig. 1. Numbers at the top of each bar indicate the statistical significance for the test of the null hypothesis that the genetic correlation coefficient is zero

The 44 lipid species that were significantly genetically correlated with T2D-related traits included 17 diacylglycerols (DAG), 17 triacylglycerols (TAG), three species each from the lysophatidylcholine and alkyllysophospahtidylcholine classes and two species each from the ceramide and cholesterol ester classes. Using the enrichment score and adjusting for 23 lipid classes, the DAG and TAG lipid classes were clearly overrepresented in the genetically correlated lipid species (Fig. 3a). We therefore examined if the total concentration of all species within these two classes was genetically correlated with T2D-related traits. We observed that both the DAG and TAG concentrations were significantly genetically correlated with each of the three T2D-related traits (Fig. 3b).

## Discussion

Our results provide compelling evidence in favor of a genetic pleiotropy between circulating levels of lipid species and T2D-related traits. Specifically, the DAG and TAG lipid classes were significantly genetically correlated with T2D and six members of the TAG class were genetically correlated with risk of T2D, presence of prediabetes as well as insulin resistance. Implied evidence in favor of a pleiotropic nexus between lipids and T2D is accumulating. Li et al. [18] have recently demonstrated using data from two, large cohorts that there is a pleiotropic association of lipid genes with blood glucose and HOMA-IR. It is noteworthy that the lipid levels and genes considered by Li et al. [18] correspond with the traditional measures of lipidemia like total serum cholesterol, serum triglycerides and high-density lipoprotein cholesterol. The fact that TAG as a lipid class was found in our study to be genetically correlated with T2D is in line with the observations of Li et al. [18].

However, value of plasma lipidomic studies lies in the refined resolution of the investigations. Our observations suggest that not all TAGs are genetically correlated with T2D and that the search for pleiotropic clues can be restricted to the more informative TAG species than the overall levels of serum triglycerides. It is also interesting that all the six TAG species genetically correlated with all the T2D-related traits had palmitate at the first position. Combined with palmitoloeate or stearate moieties, the 1-palmitoyl containing species were positively genetically correlated with all T2D-related traits. These results conceptually corroborate the growing understanding that palmitate metabolism may be partially compensated through genetic [19, 20] or epigenetic [21] mechanisms in T2D.

While the exact genetic explanation for a link between 1-palmitate containing TAG species and type 2 diabetes remains obscure, the evidence for a possible genetic link between palmitate-regulated genes and type 2 diabetes is growing. For example, over a decade ago Kelpe et al. [22] demonstrated using elegant experiments that palmitate-mediated ceramide synthesis is transcriptionally involved in the genetic control of insulin secretion. In contrast, a recent genome-wide association study [23] showed that an orchestrated control of several genes plays a part in the palmitate-associated beta-cell death. More recently, it has been posited that palmitate can moderate tool-like receptor 4 (TLR4) signaling and can thus contribute to diabetes pathogenesis through regulation of the gluconeogenic genes. In the context of insulin resistance, Yang et al. [24] have recently shown that palmitate can impair the expression of FNDC5, CTRP15 and FGF21genes in C2C12 myotubes and thus ensue insulin resistance. It has also been shown that the downstream metabolic effects of circadian rhythm disturbances (which are exerted genetically) may be triggered by palmitate fluctuations [25, 26]. These observations lend strong support for our finding that the 1-palmitate containing TAG species may be genetically correlated with risk of diabetes, prediabetes and insulin resistance. Further studies are required to localize and specifically understand the shared genetic influences on the 1-palmitate containing TG species and T2D.

Our findings of striking environmental correlations between members of the dihydroceramide and ceramide classes with T2D-related traits deserves mention. It must be realized that bivariate trait analyses partition the total phenotypic variance into shared genetic and shared environmental components. Thus, a lack of genetic correlation should be interpreted as lack of shared (pleiotropic) loci that can explain concurrent variation in two traits (e.g. between a given lipid species and a selected T2D-related trait) [14]. Therefore, the strong environmental correlations observed with dihydroceramide and ceramide species is most likely indicative of a lack of common genetic control of circulating levels of these classes of lipids and T2D.

Some limitations of our study need to be recognized. First, a potential overlap among the observed genetic correlations can be conceptualized to be consequent to a phenotypic correlation among the T2D-related traits. While this possibility cannot be ruled out, it was unlikely in our dataset for the following reasons: a) The analyses for prediabetes and HOMA-IR were restricted to individuals who did not have T2D at baseline; b) the number of lipid species genetically correlated with HOMA-IR far exceeded that genetically correlated with prediabetes; and c) not all lipid species that were significantly genetically correlated were concomitantly correlated with HOMA-IR. Second, it is possible that by excluding individuals with T2D at baseline for the analyses related to prediabetes and HOMA-IR, we may have reduced the post hoc power. Our calculations indicate that to detect a genetic correlation coefficient of 0.5 contingent on the pedigree structure of the dataset and assuming a global type I error rate of 0.05, we had a power of 88.1% in the full set of 1212 individuals. By reducing the sample size by ~15%, we found that the power was reduced to 79.2%. Thus, we believe that we had sufficient power to detect the correlation signals for all traits studied here.

## Conclusions

Taken together, these findings imply that there is a likely overlap in the set of polygenes that are associated with circulating levels of some lipid species and the T2D pathogenesis. In and of itself, statistical evidence for pleiotropy does not prove the genetic underpinnings of any biological process. However, it is a necessary first step in the direction of trying to tease apart the shared genetic influence on biologically meaningful phenotypic traits. To that end, our study is the first investigation of the potentially shared genetic influences on the plasma lipidome and T2D pathogenesis. Our results imply that urgent steps need to be taken to understand the joint genetic drivers of the plasma lipidome and T2D.

## Additional files

Additional file 1: Figure S1. Comparison of genetic (ρG) and environmental (ρE) correlation coefficient for the 44 lipid species with each T2D-related trait. (PDF 84 kb)

Additional file 2: Table S1. Biological relationships among study participants. (DOCX 12 kb)

Additional file 3: Table S2. Results of censored normal regression. (XLSX 35 kb)

Additional file 4: Table S3. Genetic correlation of plasma lipid species with T2D-related traits. (XLSX 42 kb)

Additional file 5: Table S4. Environmental correlation of plasma lipid species with T2D-related traits. (XLSX 54 kb)

## Abbreviations

HOMA-IR: Homeostatic model of assessment – insulin resistance
IFG: Impaired fasting glucose
IGT: Impaired glucose tolerance
SAFHS: San Antonio Family Heart Study
T2D: Type 2 diabetes
