# Supplementary material for: Genetic correlation of the plasma lipidome with type 2 diabetes, prediabetes and insulin resistance in Mexican American families
Source: BMC Genet. 2017 May 19;18:48. doi: 10.1186/s12863-017-0515-5 (PMC5438505; doi:10.1186/s12863-017-0515-5)

Supplementary Figure 1.

Comparison of genetic (ρ_G) and environmental (ρ_E) correlation coefficient for the 44 lipid species with each T2D-related trait. Plots show estimated genetic and environmental correlation coefficients from bivariate analyses. Blue diagonal line represents the situation where the estimated genetic and environmental correlation coefficients are equal.

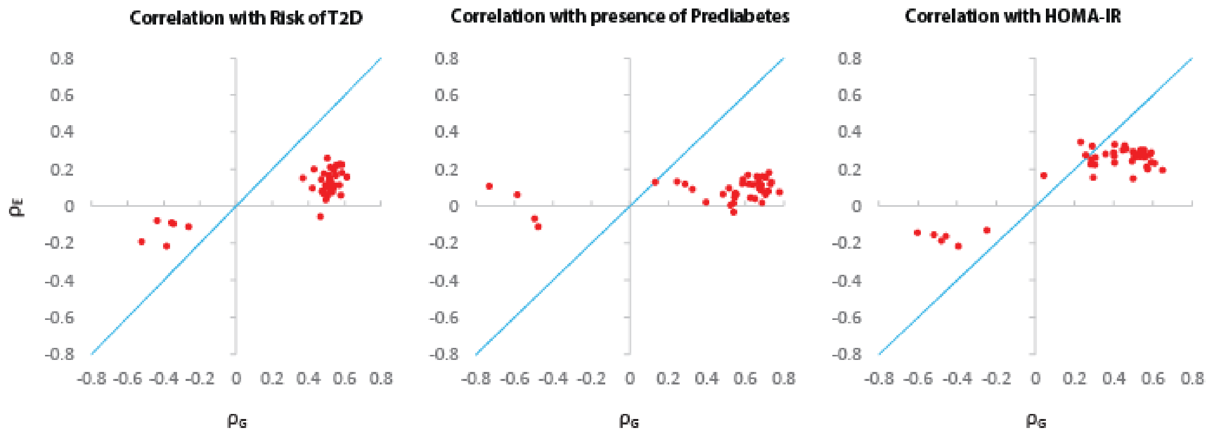

Supplement: Supplementary file 1 — Comparison of genetic (ρG) and environmental (ρE) correlation coefficient for the 44 lipid species with each T2D-related trait. (PDF 84 kb) [file 12863_2017_515_MOESM1_ESM.pdf]
