# Supplementary material for: Genetic correlation of the plasma lipidome with type 2 diabetes, prediabetes and insulin resistance in Mexican American families
Source: BMC Genet. 2017 May 19;18:48. doi: 10.1186/s12863-017-0515-5 (PMC5438505; doi:10.1186/s12863-017-0515-5)
Supplement: Supplementary file 2 — Biological relationships among study participants. (DOCX 12 kb) [file 12863_2017_515_MOESM2_ESM.docx]

Supplementary Table 1: Biological relationships among study participants.

| **Relationship** | **# pairs** |
| --- | --- |
| 3rd degree relative | 3293 |
| 4th degree relative | 2822 |
| Avuncular relatives | 2051 |
| 5th degree relative | 1204 |
| Sibling | 1098 |
| Parent-offspring | 910 |
| 6th degree relative | 316 |
| Grandparent-grandchild | 301 |
| Half siblings | 147 |
| Double first cousins | 8 |
| Monozygotic twins | 1 |
